# Cardiovascular characterisation of a novel mouse model that combines hypertension and diabetes co-morbidities

**DOI:** 10.1038/s41598-023-35680-w

**Published:** 2023-05-30

**Authors:** Arpeeta Sharma, Judy S. Y. Choi, Anna M. D. Watson, Leila Li, Thomas Sonntag, Man K. S. Lee, Andrew J. Murphy, Miles De Blasio, Geoffrey A. Head, Rebecca H. Ritchie, Judy B. de Haan

**Affiliations:** 1 Group Leader (Oxidative Stress Laboratory), Diabetic Complications Division, Baker Heart and Diabetes Institute, 75 Commercial Road, Melbourne, VIC 3004 Australia; 2 Department of Diabetes, Central Clinical School, Monash University, Melbourne, VIC Australia; 3 Drug Discovery Biology, Monash Institute of Pharmaceutical Sciences, Monash University, Parkville, VIC Australia; 4 Department of Immunology and Pathology, Central Clinical School, Monash University, Melbourne, VIC Australia; 5 Department of Physiology, Anatomy and Microbiology, La Trobe University, Melbourne, Australia; 6 Faculty of Science, Engineering and Technology, Swinburne University, Melbourne, Australia; 7 Department of Cardiometabolic Health, University of Melbourne, Parkville, Australia

**Keywords:** Biological techniques, Physiology

## Abstract

Epidemiologic data suggest that the prevalence of hypertension in patients with diabetes mellitus is ∼1.5–2.0 times greater than in matched non-diabetic patients. This co-existent disease burden exacerbates cardiac and vascular injury, leading to structural and functional changes to the myocardium, impaired cardiac function and heart failure. Oxidative stress and persistent low-grade inflammation underlie both conditions, and are identified as major contributors to pathological cardiac remodelling. There is an urgent need for effective therapies that specifically target oxidative stress and inflammation to protect against cardiac remodelling. Animal models are a valuable tool for testing emerging therapeutics, however, there is a notable lack of appropriate animal models of co-morbid diabetes and hypertension. In this study, we describe a novel preclinical mouse model combining diabetes and hypertension to investigate cardiac and vascular pathology of co-morbid disease. Type 1 diabetes was induced in spontaneously hypertensive, 8-week old, male Schlager (BPH/2) mice via 5 consecutive, daily injections of streptozotocin (55 mg/kg in citrate buffer; i.p.). Non-diabetic mice received citrate buffer only. After 10 weeks of diabetes induction, cardiac function was assessed by echocardiography prior to post-mortem evaluation of cardiomyocyte hypertrophy, interstitial fibrosis and inflammation by histology, RT-PCR and flow cytometry. We focussed on the oxidative and inflammatory stress pathways that contribute to cardiovascular remodelling. In particular, we demonstrate that markers of inflammation (monocyte chemoattractant protein; MCP-1), oxidative stress (urinary 8-isoprostanes) and fibrosis (connective tissue growth factor; CTGF) are significantly increased, whilst diastolic dysfunction, as indicated by prolonged isovolumic relaxation time (IVRT), is elevated in this diabetic and hypertensive mouse model. In summary, this pre-clinical mouse model provides researchers with a tool to test therapeutic strategies unique to co-morbid diabetes and hypertension, thereby facilitating the emergence of novel therapeutics to combat the cardiovascular consequences of these debilitating co-morbidities.

## Introduction

Diabetes and hypertension are two interlinked co-morbidities that significantly increase the incidence of cardiovascular complications. Approximately 20% of hypertensive patients develop diabetes and up to 85% of diabetic patients develop hypertension, depending on individual factors such as age and genetics^1,2^. This results in a two–threefold increased risk of developing coronary artery disease, cardiomyopathy, myocardial infarction and congestive heart failure. Furthermore, the prevalence of diabetes and hypertension is increasing at an alarming rate globally^3^. Diabetic patients develop a distinct form of cardiomyopathy characterised by structural and functional cardiac alterations, including impairment of diastolic function, cardiac hypertrophy and fibrosis, as well as cardiomyocyte cell death^4,5^. Current standard of care for patients with hypertension and diabetes includes drugs targeting the renin-angiotensin system (eg. ACEi, ARBs), statins, calcium-channel antagonists and thiazide diuretics. Diabetic patients with co-morbid hypertension usually require combination therapy to achieve their blood pressure goals, often with multiple antihypertensive agents and at higher dosages^6–9^. Additionally, in patients with hypertension and diabetes, blood pressure lowering treatment has to commence at the lower threshold of ≥ 130/80 mm Hg instead of the recommended threshold of ≥ 140/90 mm Hg for the general adult population^8,9^. Therefore, there remains an urgent unmet clinical need to develop safe and effective therapies to target cardiovascular complications that arise from these co-morbidities. In this respect, animal models remain indispensable for validating and optimizing more potent and specific drug targets and are a critical tool for bench-to-bedside drug discovery.

A limited number of animal models of co-morbid diabetes and hypertension exist that exhibit cardiac remodelling and recapitulate human disease^10,11^. However, limitations inherent in model design limit their usefulness for drug discovery. In most instances, the level of hypertension achieved is modest, of short duration, and hypertension needs to be chemically induced. Furthermore, many models involve rats which limits the use of genetic manipulation.

The most widely used co-morbid model involves short-term angiotensin II (Ang-II) infusion^12^ into streptozotocin (STZ)-induced diabetic rats or mice. Hypertension is induced via a single osmotic mini-pump delivering Ang-II via 14- or 28-day pumps, although 14-day minipumps are mostly recommended for use in mice^12,13^. The hypertension achieved via minipump infusion is moderate, driven by vasoconstrictor responses, and prone to decreases in blood pressure as vasoconstrictor action diminishes^12,14^. Timely replacement and/or removal of minipumps is critical to prevent pump swelling and concentrated salt leakage. These limitations restrict long-term usage to study chronic complications of co-morbid disease.

An alternate approach is the spontaneously hypertensive rat (SHR) combined with STZ-induced diabetes^15^. The STZ-SHR model is well characterised and develops a hyperglycaemic syndrome, with biochemical and morphological changes representative of Type 1 diabetes combined with hypertension. In particular, the STZ-SHR model develops endothelial dysfunction and adverse cardiac remodelling, with left ventricular hypertrophy and dilatation, however cardiac fibrosis is not observed^16^. The obese Zucker rat eliminates the need for STZ administration and is characterised by the simultaneous occurrence of obesity, hyperglycaemia, hyperinsulinemia, hyperlipidaemia and moderate hypertension, closely mimicking a Type 2 diabetic profile. Cardiovascular changes have been observed in these obese rats, including cardiac oxidative stress and inflammation, diastolic dysfunction and cardiac remodelling^17,18^. Other rat models include the Dahl-salt sensitive hypertensive rat rendered diabetic by STZ, where renal pathology has been extensively investigated^19–21^. However, hypertension also develops in aged non-diabetic rats, making it difficult to decipher the link between aging, hypertension and diabetes^22^.

There are relatively few spontaneously hypertensive mouse strains available for hypertensive research, which are advantageous over rat models due to tools available for genetic manipulation, ease of breeding and cost-effectiveness of housing. The Schlager inbred hypertensive mouse model has gained significant interest, where the mechanisms driving the high blood pressure have been extensively characterised^23^. These genetically hypertensive mice are one of three lines, namely BPL/1 (Blood Pressure Low), BPH/2 (Blood Pressure High), and BPN/3 (Blood Pressure Normal), which were selectively bred based on their blood pressure^24^. BPH mice have a spontaneous form of hypertension driven by an overactive sympathetic nervous system (SNS), which is apparent from 6 weeks of age based on tail-cuff and radio-telemetry measurements^23,25^. Systolic blood pressure of BPH mice differs from their BPN counterparts by ~ 24% at 14–21 weeks of age^23,26^. In a recent study by our group, we induced diabetes in the Schlager BPH mice via STZ and assessed the effects of diabetes and hypertension on renal structure and function, renal catecholamines and oxidative stress. Indeed, in this co-morbid model of diabetes and hypertension, there were overt changes in albuminuria and renal oxidative stress associated with a compromised anti-oxidant system^26^. In the present study, we characterised the cardiac and vascular pathology of the diabetic BPH mouse, with a particular focus on oxidative stress and inflammatory pathways that contribute to cardiovascular remodelling. Our study establishes a novel co-morbid mouse model of diabetes and hypertension that offers ease of model generation, maintenance of diabetes and hypertension for studies of longer duration, and facilitates investigations of co-morbid disease on cardiovascular and renal endpoints.

## Methods

### Animals

All experiments were approved by the Alfred Medical Research Education Precinct (AMREP) animal ethics committee. Male hypertensive (BPH/2J) and normotensive (BPN/3J) Schlager mice were bred and housed at the AMREP animal facility. At 8 weeks of age, mice were randomly assigned to a non-diabetic or diabetic experimental group. Diabetes was induced by 5 consecutive daily IP injections of STZ (Sigma Aldrich) at 55 mg/kg/day in citrate buffer (pH 4.5), while non-diabetic mice received citrate buffer only. This concentration of STZ, when administered over 5 days, is at the lower end of published STZ dosage regimens and was chosen to limit cardiac and renal toxicity^27^. Mice had unrestricted access to food (normal chow diet) and water and were maintained on a 12 h light and 12 h dark cycle. Non-fasted blood glucose and body weights were recorded on a weekly basis to confirm diabetic status. At endpoint, 10 weeks after the induction of diabetes, mice were killed and the heart and aortae were collected for analysis. The study is reported in accordance with ARRIVE guidelines.

### Cardiac functional assessments by echocardiography

Functional echocardiography was performed by the Baker Institute Preclinical Cardiology Microsurgery and Imaging Platform. Two-dimensional (2D) parasternal, M-mode and Doppler flow echocardiograms were obtained on a Philips iE33 ultrasound system with 15 MHz linear (M-mode) and 12 MHz sector (Doppler) transducers (Koninklijke Philips©, Amsterdam, Netherlands). Echocardiograms were performed in anesthetized mice (3–4.5% isoflurane initially and 1.7% isoflurane during the procedure) at endpoint. Left ventricular (LV) filling was assessed using transmitral Doppler; the ratio of the initial (E) and second (A) blood flow velocities (E/A ratio), deceleration time and isovolumic relaxation time (IVRT) were measured. Echocardiograms were analysed using the Vevo LAB™ software (v3.2.5; FUJIFILM VisualSonics Toronto, ON, Canada). Five measurements for each parameter were obtained and averaged per mouse. A reduced E/A ratio, increased E/e’ ratio and prolonged IVRT and deceleration times were used as an indicator of diastolic dysfunction. All echocardiographic imagery was acquired by a single operator and analysed using previously standardized guidelines with quality control for analysis of echocardiography in the mouse^28^.

### Blood sampling and tissue collection

Heparinized blood, collected after puncture of the right ventricle, was centrifuged to obtain plasma samples. Peripheral fat was removed from aortae in Krebs buffer and cut into two sections. Thoracic aortae were mounted on a wire myograph (Danish Myo Technology) for vascular reactivity analysis while the arch and abdominal aortae were snap-frozen and stored at − 80 °C for RNA extraction. Hearts were removed and weighed. The left ventricle was further divided into three sections. The base of the heart was fixed in 10% neutral-buffered formalin and paraffin-embedded for histology. The middle portion was frozen in OCT for histology while the apex was snap-frozen in liquid nitrogen and stored at – 80 °C for gene expression analysis.

### Vascular function

To determine if the combination of diabetes and hypertension in our mouse model alters vascular function, vascular reactivity studies were performed as published previously^29^. Briefly, thoracic aortae were cut into 4 mm segments and mounted on two L-shaped metal prongs. Aortae were equilibrated for 30 min at a resting tension of 5 mN. All aortae were then exposed to high potassium physiological salt solution (KPSS) to determine viability. Next, cumulative concentration-responses to acetylcholine (ACh; 1 nM to 100 µM) were recorded in aortae pre-constricted to approximately 50% KPSS with phenylephrine (PE). All vasorelaxation responses are presented as percentage relaxation of the pre-constriction response. Additionally, a concentration–response curve to PE (1 nM to 100 µM) was performed to assess vascular contractility. The variable slope sigmoidal concentration–response curves to all agonists for each mouse were calculated and plotted using GraphPad Prism (version 8.0).

### Histology

Cardiac fibrosis was assessed by picrosirius red staining to quantify deposition of collagen fibres. In brief, 4-µm paraffin-embedded heart sections were dewaxed, fixed in 10% normal buffered formalin (NBF) and stained with picrosirius red (99% Picric acid, 1% Sirius Red) for 1 h. Sections were then differentiated in 0.01 M hydrochloric acid, washed and dehydrated before mounting with DePex. Stained sections were imaged on the Olympus BX43 light microscope (Olympus Life Science, Waltham, MA, USA) at 200X magnification, using the cellSens software (Olympus Life Science, Waltham, MA, USA). 10 images per section were obtained under bright-field and polarised-light to differentiate between type I and type III collagen fibres. Collagen deposition was analysed using ImagePro software.

To assess cardiomyocyte size, haematoxylin and eosin was used to stain the nuclei and cytoplasm of cardiac cells, respectively. Briefly, paraffin-embedded heart sections were dewaxed, rehydrated and stained with Mayer’s Haematoxylin for 6 min. After washing in water, sections were differentiated in Scott’s tap water for 30 s followed by alcoholic Eosin Y solution (99% ethanol, 1% eosin, 0.1% acetic acid) for 2 min as a counterstain. Sections were then dehydrated and mounted with DePex mounting media. Stained sections were imaged using an Olympus BX43 light microscope at 400X magnification in bright-field. 10 images per section were randomly taken around the LV lumen, using the cellSens software (Olympus Life Science, Waltham, MA, USA). Images were then analysed on the ImageJ/Fiji software by tracing around the perimeter of randomly selected cardiomyocytes to determine cell area. The diameters across the shortest, cross-sectional axis of the same cells were also measured to determine cell width. At least 100 cardiomyocytes were measured and averaged per sample, across the 10 images^4^.

### Immunohistochemistry

Nitrotyrosine expression, an indicator of oxidative stress, and CD68 expression, a macrophage specific marker, was determined by immunohistochemistry^29^. In brief, paraffin-embedded heart sections were dewaxed and endogenous peroxidases were inactivated with 3% H_2_O_2_ in Tris-buffered saline (pH 7.4). Sections were then incubated with a serum blocking agent and a biotin-avidin blocking kit (Vector Laboratories) for 1 h and then with respective primary antibodies overnight at 4 °C. Secondary antibody, biotinylated anti-rabbit immunoglobulin or the biotinylated anti-rabbit secondary antibody was then added for 30 min, followed by horseradish peroxidase–conjugated streptavidin (1:500), and incubated for 3 min in 3,3′-diaminobenzidine tetrahydrochloride and counterstained with hematoxylin. Images were visualized under light microscopy and quantitated using Image Pro Plus.

### Gene expression

Total RNA was extracted after homogenization of snap-frozen left ventricles (LV) and aortae, and gene expression was analysed by quantitative RT-PCR (qRT-PCR) as described previously^29^. Genes to be analysed were chosen based on the role they play in cardiovascular function, oxidative stress and inflammation. In the aortae, inflammatory genes (tumour necrosis factor-α (TNF-α), monocyte chemotactic protein-1 (MCP-1) and interleukin-1β (IL-1β)) and oxidative stress genes (Nox1, Nox4 and p47phox) were analysed. In LV samples, markers of cardiac function (atrial natriuretic peptide (NPPA), brain natriuretic peptide (NPPB) and β-myosin heavy chain (β-MHC)), cardiac fibrosis (connective tissue growth factor (CTGF), transforming growth factor-β (TGF-β)), cardiac antioxidants (Nrf2, NQO1 and HO-1) and cardiac inflammation (MCP-1 and IL-1β) were analysed. mRNA sequences are listed in Supplementary Table 1.

### Flow cytometry

Leukocytes including B cells, T cells, neutrophils and monocytes, were identified using flow cytometry as previously described^30^. Blood was collected via cardiac puncture into heparinised tubes, which were immediately incubated on ice. The spleen was minced, flushed through a 40 µm cell strainer and centrifuged at 300 g for 5 min at 4 °C. Bone marrow was cleaned and flushed to collect cells. All subsequent steps were performed on ice. Red blood cells were lysed (BD pharm Lyse; BD Biosciences), and WBCs were centrifuged at 300 g for 5 min at 4 °C, washed, and resuspended in Hanks buffered saline solution (HBSS, 0.1% bovine serum albumin (BSA) w/v, 5 mM EDTA). Cells from the blood, bone marrow and spleen were stained with a cocktail of antibodies against CD45-PB, Ly6-C/G-PerCP-Cy5.5 (BD Biosciences), CD115-APC (eBioscience), siglec F-PE (eBioscience), CD3-FITC (BD Biosciences) and CD19-APC (eBioscience). Monocytes were identified as CD45^+^CD115^hi^; neutrophils were identified as CD45^+^CD115^lo^Ly6-C/G^hi^ (Gr-1). T cells were identified CD45^+^CD115^lo^Ly6-C/G^lo^CD3^+^ and B cells as CD45^+^CD115^lo^Ly6-C/G^lo^CD19^+^. All samples were run on a BD LSR Fortessa and analysed using FlowJo 7.6 software (Treestar, USA).

Cells from the left ventricles were enzymatically-dissociated using HBSS supplemented with 2% FBS, 0.9 mM CaCl_2_, Collagenase 1A (10 mg/ml), DNAase1 (10 mg/ml) and Dispase-2 (10 U/ml). Briefly, the heart was perfused with PBS and the left ventricular apex was separated and cut into small pieces and incubated in the enzymatic solution for 30 min at 37 °C with mild agitation. The resulting single-cell suspension was resuspended in HBSS, and cells were stained for 30 min on ice with anti-mouse CD45-PB, F4/80-PE, CD11b-BV786, CD31-FITC and live/dead marker (AF700). Cells were analysed on a BD FACS Canto II flow cytometer (Becton Dickinson, Franklin Lakes, NJ, USA) using FlowJo 7.6 software (Treestar, USA). Endothelial cells were identified as CD31^+^CD45^-^ and macrophages were identified as CD11b^+^F4/80^+^.

### Statistics and blinding

All data are expressed as mean ± standard error of mean (SEM). Comparisons between groups were analysed using a two-way ANOVA, followed by Tukey’s post-hoc test. Analysis was conducted using GraphPad Prism (v8.0 GraphPad Software, La Jolla, CA, USA). A *P*-value of < 0.05 was considered statistically significant. Researchers were blinded to experimental groups, and results were decoded after analysis.

### Ethical approval

All animal experiments were approved by the Alfred Medical Research and Education Precinct animal ethics committee (Ethics number: E1895/2018/B), Melbourne, Australia. All investigations were performed in accordance with the National Health and Medical Research Council (NHMRC; Australia) guidelines.

## Results

### Mortality data and metabolic, cardiac and blood pressure characteristics

STZ treatment can result in a high mortality rate dependent on species, dosage and diet^27^. We have specifically used a low-dose regimen to limit toxicity and determined the mortality rate of our 3 cohorts of mice (see Supplementary Fig. 6 for a detailed breakdown of numbers). The overall mortality rate was ~ 8% (6/78). The rate of resistance to becoming diabetic was ~ 16% (12/78).

**Figure 1:**
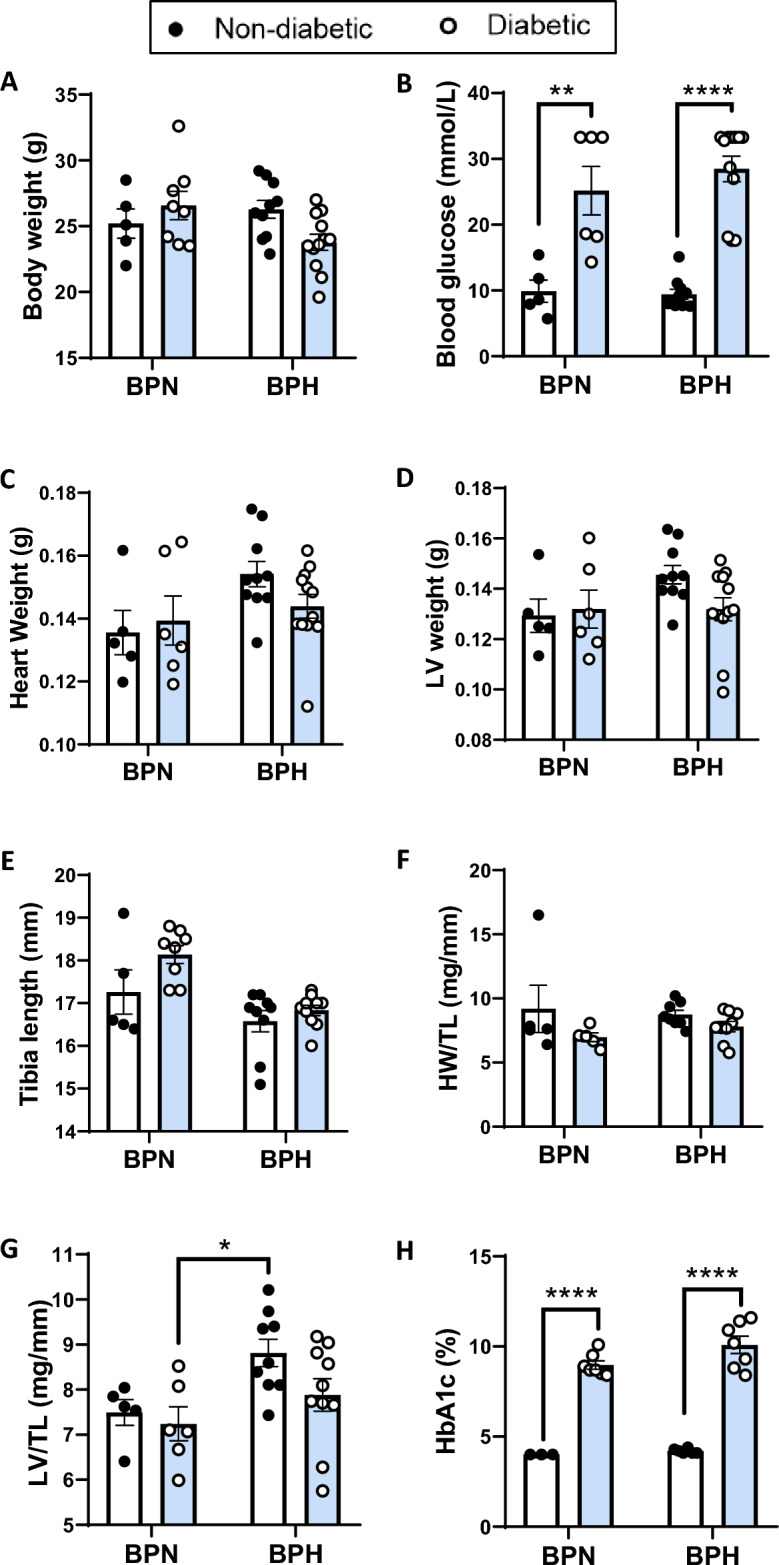
Basic characteristics of mice. (**A**) Body weight (g), (**B**) Blood glucose (mmol/L), (**C**) Heart weight (g), (**D**) Left Ventricular (LV) weight (g), (**E**) Tibial length (mm), (**F**) Heart weight: Tibial length ratio (HW/TL; mg/mm), (**G**) LV weight to tibial length ratio (mg/mm) and (**H**) Glycated haemoglobin (HbA1c) levels at the end of the study. A high blood glucose reading was given a value of 33.3 mmol/L. A below detection reading for HbA1c levels was given a value of 4.0%. *P < 0.05, **P < 0.01 and ****P < 0.0001 as indicated. Bars represent mean ± SEM, with individual values plotted.

We have previously reported, using the same cohort of animals investigated in this study, that water intake and urine output was elevated in the diabetic mice (*P* < 0.001)^26^. Furthermore, our published data showed that mean arterial pressure and systolic blood pressure was significantly higher in non-diabetic and diabetic BPH mice as compared to BPN mice at endpoint. Mean arterial pressure was similar in diabetic and non-diabetic BPH groups indicating that diabetes per se has no impact on blood pressure^26^. In the current study, we report that the diabetic BPH group demonstrated a tendency towards lower body weight compared to non-diabetic BPH mice as well as BPN mice, however this did not reach significance (*P* = 0.095; Fig. 1A). Blood glucose concentrations and HbA1c, measured at endpoint, were significantly higher in the diabetic BPN and BPH mice compared to their non-diabetic counterparts (*P* < 0.001, Fig. 1B,H). There was a tendency towards increased heart weight (Fig. 1C, *P* < 0.1) and significant increases in LV weight when normalised to tibia length (Fig. 1D,G, *P* < 0.05) in non-diabetic BPH mice compared to non-diabetic BPN mice. There was no difference between LV weight in the diabetic BPH mice compared to non-diabetic BPH mice (Fig. 1G). Additionally, there was no difference in tibia length and heart weight normalized to tibia length (Fig. 1E,F).

### Oxidative stress

**Figure 2:**
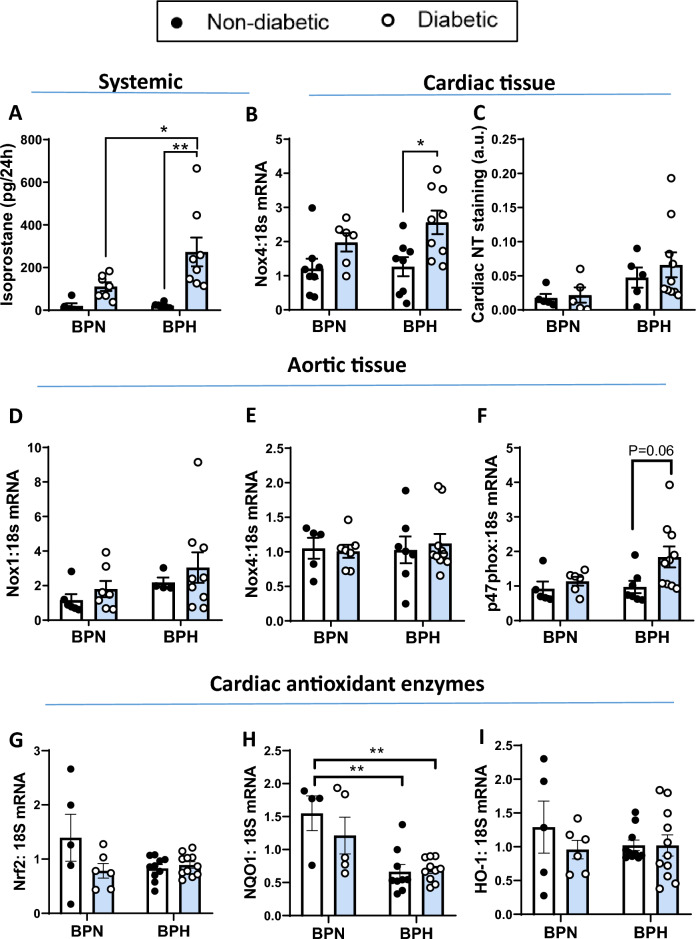
Oxidative stress analysis. (**A**) Urinary isoprostanes concentration over a 24 h period. (**B**) Cardiac *Nox4* gene expression and (**C**) Quantificaiton of cardiac nitrotyrosine (NT) staining. Aortic tissue (**D**) *Nox1*, (**E**) *Nox4* and (**F**) *p47phox* gene expression levels. (**G**–**I**) Cardiac (**G**) Nrf2, (**H**) NQO1 and (**I**) HO-1 antioxidant gene expression levels. Gene expression was determined by qRT-PCR and expressed relative to non-diabetic BPN mice. *P < 0.05 and **P < 0.01 as indicated. Bars represent mean ± SEM with individual values plotted. a.u = arbitrary units.

Systemic oxidative stress was measured via urinary 8-isoprostanes. Hypertension alone did not affect urinary 8-isoprostane levels, but diabetes in normotensive and hypertensive mice increased urinary 8-isoprostane levels, with higher levels observed in the diabetic BPH mice (*P* < 0.05 vs diabetic BPN, Fig. 2A). In LV tissue, an increase in *Nox4* gene expression was seen in diabetic BPH compared to non-diabetic BPH mice (*P* < 0.05; Fig. 2B). There was no difference in cardiac NT staining between groups (Fig. 2C). In the vasculature, neither *Nox1* nor *Nox4* gene expression was affected by either hypertension or diabetes (Fig. 2D,E). There was a tendency towards upregulation of vascular p47phox in the diabetic BPH mice (*P* = 0.06; Fig. 2F). Additionally, in LV tissues, gene expression of the master regulator of antioxidant gene expression, Nrf2, and downstream antioxidants NQO1 and HO-1 were analysed (Fig. 2G–I). Gene expression of NQO1 was significantly reduced in non-diabetic and diabetic BPH mice as compared to BPN mice (Fig. 2H), whilst expression of Nrf2 and HO-1 were unaffected by either hypertension or diabetes (Fig. 2G,I).

### Vascular inflammation and function

**Figure 3:**
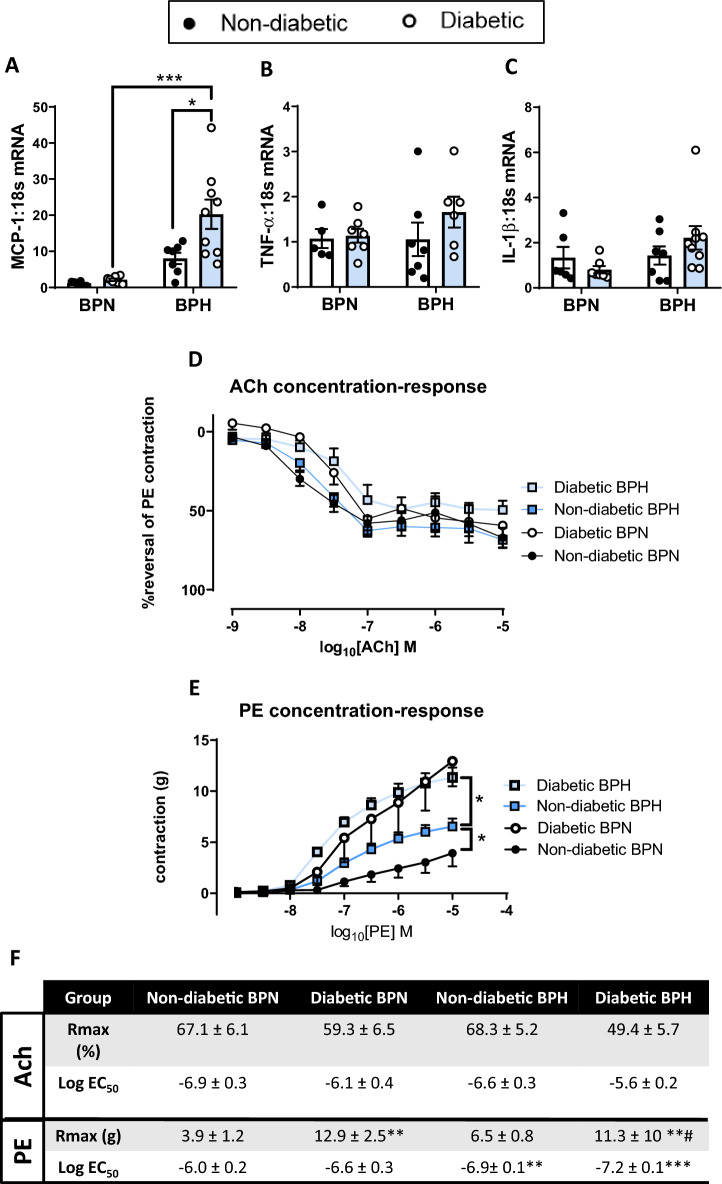
Vascular inflammation and vascular function. Aortic tissue. (**A**) *MCP-1*, (**B**) *TNF-α* and (**C**) *IL-1β* gene expression levels determined by qRT-PCR and expressed relative to non-diabetic BPN mice. (**D**) Percentage reversal of phenylephrine (PE)-induced contraction in response to increasing concentrations of acetylcholine (ACh) and (**E**) vascular contraction in response to PE. (**F**) R_max_ and Log EC_50_ values for ACh and PE concentration–response curves from (**D**) and (**E**). *P < 0.05 and ***P < 0.001 as indicated. #*P* < 0.05 vs non-diabetic BPH. Bars represent mean ± SEM with individual values plotted.

Gene expression of the pro-inflammatory cytokine, *MCP-1*, was eightfold higher in aortas of non-diabetic BPH mice as compared to non-diabetic BPN mice (Fig. 3A). Diabetes alone did not have an effect on vascular *MCP-1* gene expression, however, the combination of diabetes and hypertension further augmented *MCP-1* gene expression in the diabetic BPH mice as compared to non-diabetic BPH mice (*P* < 0.05, Fig. 3A). Additionally, there was a tendency towards increased *TNF-α* and increased *IL-1β* gene expression (*P* < 0.1) in the diabetic BPH mice as compared to non-diabetic BPH mice as well as diabetic BPN mice (Fig. 3B,C). Vascular function was assessed by acetylcholine-induced endothelium-dependent vasorelaxation and phenylephrine-induced contraction. In this model, neither diabetes, hypertension or the combination of both diabetes and hypertension had an impact on acetylcholine-induced vasorelaxation (Fig. 3D,F). Conversely, non-diabetic BPH mice demonstrated increased vascular contractility in response to phenylephrine as compared to non-diabetic BPN. Both diabetic mice groups displayed further augmentation of vascular contractility as compared to non-diabetic BPN and BPH mice (Fig. 3E,F).

### Cardiac function and fibrosis

**Figure 4:**
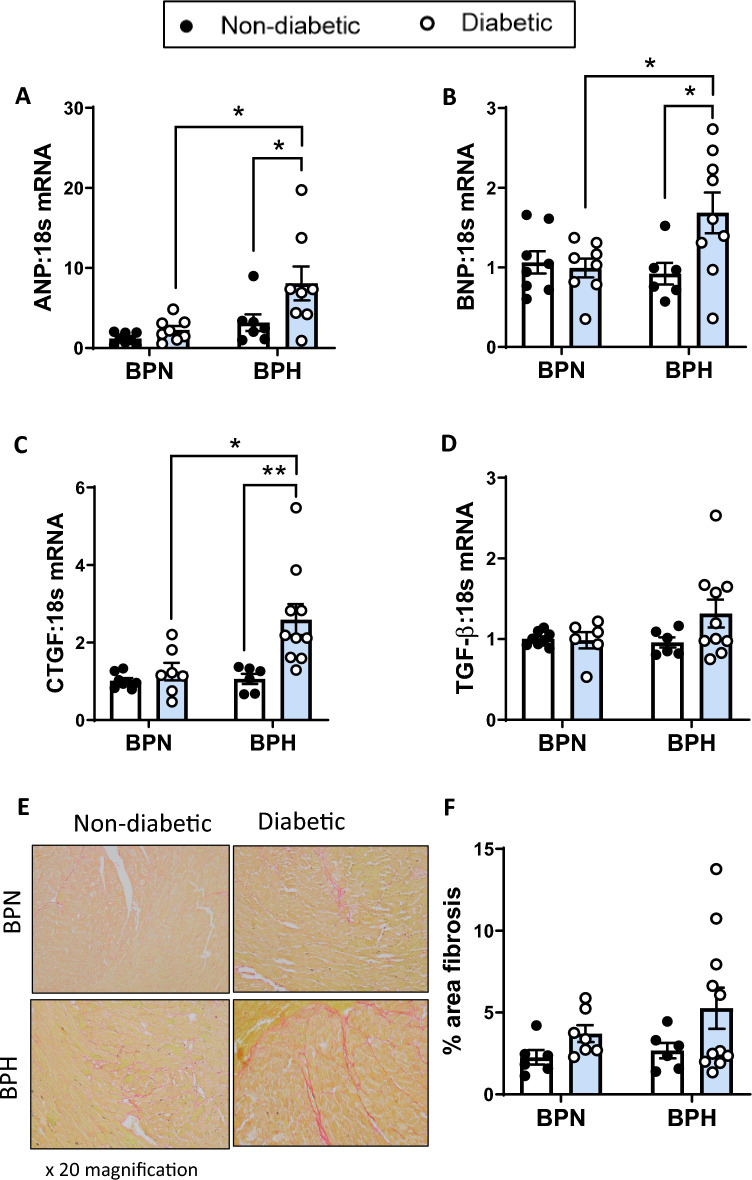
Markers for cardiac function and cardiac fibrosis. (**A**) *ANP*, (**B**) *BNP*, (**C**) *CTGF* and (**D**) *TGF-β* gene expression levels determined by qRT-PCR and expressed relative to non-diabetic BPN mice. (**E**) Representative images and (**F**) quantification of picrosirius red staining in cardiac tissues. *P < 0.05 and **P < 0.01 as indicated. Bars represent mean ± SEM with individual values plotted.

LV gene expression of markers of cardiac function, *NPPA* and *NPPB*, were both upregulated in the diabetic BPH mice as compared to diabetic BPN and non-diabetic BPH mice (*P* < 0.05; Fig. 4A,B), however, *β-MHC* gene expression was unaffected by diabetes or hypertension (Supplementary Fig. 1A). Cardiomyocyte hypertrophy as assessed by H&E analysis demonstrated that non-diabetic BPH mice have larger cardiomyocyte area and cardiomyocyte width as compared to non-diabetic BPN mice (*P* < 0.05, Supplementary Fig. 1B and 1C). Interestingly, diabetic BPH mice exhibited a reduction in cardiomyocyte area and width as compared to control BPH mice (Supplementary Fig. 1B and 1C). Cardiac fibrosis was assessed by analysing LV fibrotic gene expression as well as picrosirius red staining for collagen fibres. Gene expression of the pro-fibrotic gene, *CTGF*, was significantly increased by 2.5-fold in diabetic BPH mice as compared to diabetic BPN and non-diabetic BPH mice (Fig. 4C). *TGF-β* showed no difference in expression between the experimental groups (Fig. 4D). Histological picrosirius staining demonstrated a tendency towards increased collagen deposition in the diabetic BPH mice (Fig. 4E,4F).

### Cardiac inflammation

**Figure 5:**
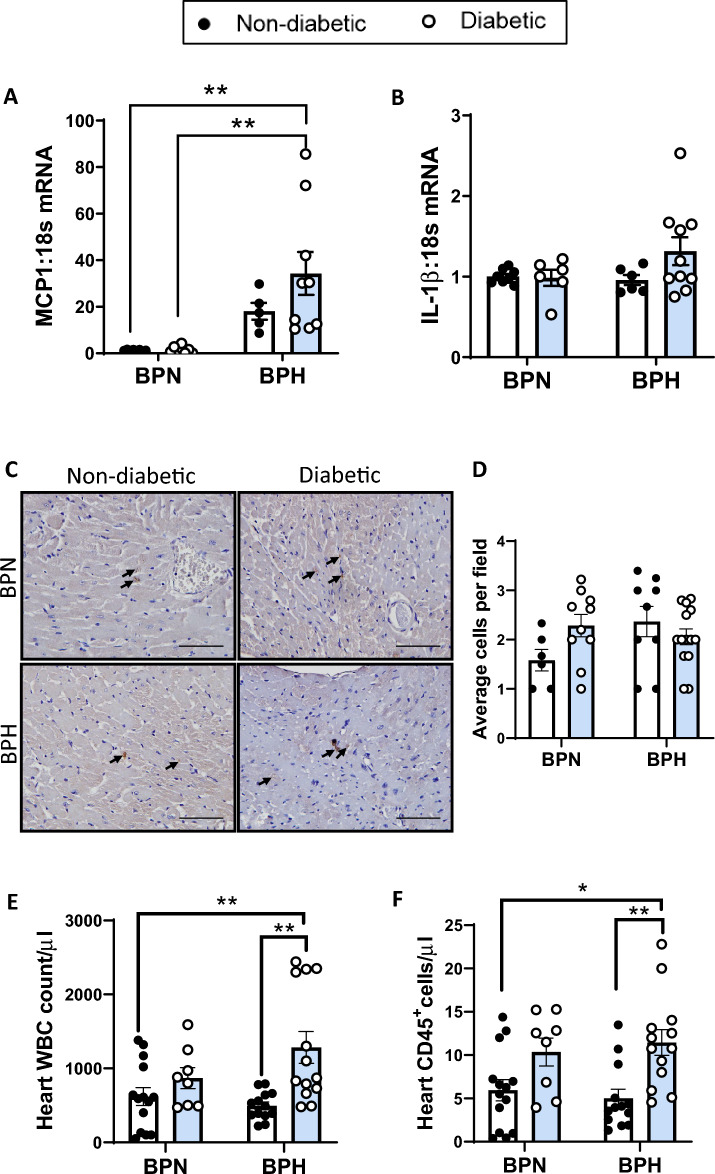
Cardiac inflammation. (**A**) *MCP-1* and (**B**) *IL-1β* gene expression levels determined by qRT-PCR and expressed relative to non-diabetic BPN mice. (**C**) Representative images and (**D**) quantification of CD68 immunostaining in cardiac tissue. Arrows demonstrate positive staining of macrophages. Scale bars represent 100 µm. (**E**) Cardiac white blood cell (WBC) determined by Hemavet technology. (**F**) Quantified abundance of CD45 + leukocytes in the heart by Flow Cytometry. Bars represent mean ± SEM with individual values plotted. *P < 0.05 and **P < 0.01 as indicated.

Cardiac inflammation was assessed by the gene expression of the pro-inflammatory chemokine (*MCP-1*) and cytokine (*IL-1β*) as well as CD68 staining for macrophages. Similar to the vasculature, *MCP-1*, was elevated in the hearts of non-diabetic BPH mice as compared to non-diabetic BPN mice (Fig. 5A). Diabetes alone did not influence cardiac *MCP-1* gene expression, however, the combination of diabetes and hypertension further augmented MCP-1 gene expression in diabetic BPH mice as compared to diabetic BPN mice (*P* < 0.05, Fig. 5A). Additionally, there was a tendency towards increased *IL-1β* gene expression in diabetic BPH mice as compared to the diabetic BPN and non-diabetic BPH groups (Fig. 5B). Macrophages were counted as CD68 positive cells per field and averaged across 5–6 fields. Macrophage counts (averaged over 6–8 images per heart) were consistent between all groups of mice (Fig. 5C,D).

### Leukocyte trafficking

Leukocyte trafficking was analysed by flow cytometry measuring CD45+ leukocytes, monocyte/macrophage markers, neutrophils and non-myeloid populations in the blood, spleen, bone marrow and heart. Within the heart, white blood cells (WBCs) were significantly elevated in diabetic BPH mice compared to non-diabetic BPN, diabetic BPN and non-diabetic BPH mouse groups (*P* < 0.01; Fig. 5E). Similar changes in CD45^+^ leukocytes were observed across all heart groups, with elevated levels observed in the diabetic BPH group versus the non-diabetic BPH group (*P* < 0.05, Fig. 5F). F4/80 macrophage counts and CD31+ endothelial cells were comparable between heart groups with no significant changes noted (Supplementary Fig. 2A,B). In the spleen, there was an increase in WBCs and CD45+ leukocyte counts in the non-diabetic BPH groups as compared to the BPN mouse groups (Supplementary Fig. 3A,B). Ly6C^hi^ monocytes and neutrophils were significantly elevated in the non-diabetic and diabetic hypertensive BPH mouse demonstrating increased cell counts as compared to their non-diabetic and diabetic BPN counterparts (Supplementary Fig. 3C,D). In the blood, both WBC counts and CD45+ cell counts demonstrated a tendency towards lower counts in both non-diabetic and diabetic BPH groups compared to BPN groups, (Supplementary Fig. 4A,B). However, these changes were not statistically significant. Furthermore, negligible changes were observed across treatment groups for both Ly6C^hi^ monocytes and neutrophils. B cells were considerably lower in both non-diabetic and diabetic BPH groups compared to non-diabetic and diabetic BPN groups (*P* < 0.001; Supplementary Fig. 4E). In the bone marrow, both WBC count and CD45^+^ leukocytes were significantly elevated in the diabetic BPN group compared to all other groups (Supplementary Fig. 5A,B). Similarly, significantly elevated Ly6C^+^ and neutrophil cell counts, as well as T cell counts, were observed in the diabetic BPN groups (Supplementary Fig. 5C–5F).

### Echocardiography

**Figure 6:**
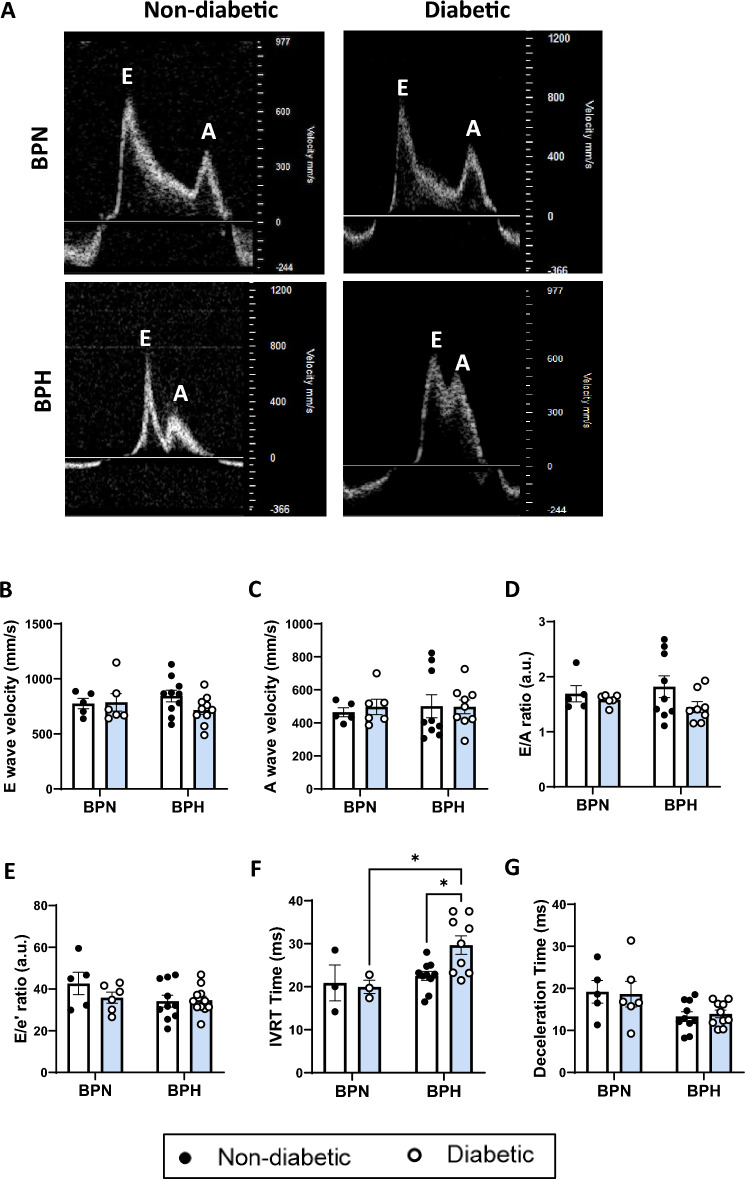
Diastolic Function measured with Doppler Echocardiography. (**A**) Representative images of MV doppler echocardiograms show reduced E-waves and E/A ratio in diabetic BPH mice (y axis = velocity [mm/s], scale bar = 10 ms). (**B**) Peak E-wave, (**C**) Peak A-wave, (**D**) E/A ratio and (**E**) E/e’ ratio (a.u. = arbitrary units). (**F**) Isovolumic relaxation time (IVRT) and (**G**) Deceleration time (ms). *P < 0.05 as indicated. Bars represent as mean ± SEM with individual values plotted.

LV diastolic function was assessed using transmitral and tissue Doppler echocardiography at endpoint. There was no overall difference in the markers of diastolic function between groups, i.e. the peak E-wave, A-wave, E/A ratio and E/e’ ratio between groups (Fig. 6A–E). However, diabetic BPH mice showed a tendency to have reduced E-wave velocities and E/A ratios (Fig. 6B,D). Diabetic BPH mice had prolonged isovolumic relaxation time (IVRT) (*P* = 0.05, Fig. 6F), which indicates LV relaxation impairment due to delayed opening of the mitral valve. However, the deceleration time of diabetic BPH mice did not differ from non-diabetic BPH mice (Fig. 6G).

## Discussion and conclusion

Diabetic patients with co-morbid hypertension are at increased risk of developing complications, which include atherosclerosis, coronary artery disease, peripheral nerve damage and kidney disease. Indeed, the underlying molecular mechanisms, which include an increase in oxidative stress, inflammation and fibrosis, are also implicated in cardiac and vascular remodelling and dysfunction in hypertension. To date, limited animal models exist that facilitate co-morbid studies. This study characterises a preclinical mouse model that combines diabetes and hypertension by inducing Type 1 diabetes in the spontaneously hypertensive Schlager mouse and demonstrates that these co-morbidities result in an exaggeration of cardiovascular pathology and function. In particular, markers of inflammation (MCP-1), oxidative stress (urinary 8-isoprostanes) and fibrosis (CTGF) are markedly increased, while diastolic dysfunction as indicated by prolonged IVRT is observed in these mice. There are several models of diabetes and hypertension, mostly rat models, that are widely used in cardiovascular research that emulate disease processes and pathogenesis. However, mouse models that combine both co-morbidities are limited. The spontaneous and sustained nature of hypertension achieved in BPH mice gives this mouse model a unique advantage over experimentally-induced hypertensive models that require extra interventions to initiate and maintain the hypertensive phenotype.

In this study, we show that administration of low-dose STZ led to increased thirst, increased urine output and consistent hyperglycaemia, all of which are prominent clinical features of Type 1 diabetes^26,31^. Co-morbid hypertension did not have an effect on these physiological characteristics of the mice. With regards to blood pressure, BPH mice are hypertensive in both the light (inactive) and dark (active) phases, as well as over 24 h^26^, with diabetic and hypertensive animals having a significantly greater mean arterial pressure and systolic blood pressure than diabetic normotensive mice as measured by telemetry^23^. This occurs despite equivalent fluid perturbations from diabetes-associated polydipsia and polyuria.

Left ventricular diastolic dysfunction is one of the first discernible manifestations of diabetic cardiomyopathy^5,32^. We observed a prolonged IVRT, indicative of impaired cardiac relaxation under diabetic and hypertensive conditions. The IVRT represents the period between aortic valve closure and mitral valve opening^33^. Although not routinely quantified in human studies, IVRT is a sensitive marker of LV diastolic function and well-characterised in diabetic and chronic pressure-overload models^34^. A consequence of impaired isovolumic relaxation is slower early-ventricular filling. This is reflected by the lower E-waves in diabetic BPH mice, albeit a non-significant trend, when compared with control BPH mice. Interestingly, the A-wave appeared to be unaffected, which suggests that there was no compensatory increased filling during late-diastole. As a result, the tendency for diabetic BPH mice to have a reduced E/A ratio was largely driven by decreased E-waves. Although the E/A ratio is one of the most commonly used parameters, it should be interpreted with caution as it can be influenced by myocardial relaxation, ventricular stiffness and left atrial pressure^34^. Generally, the E/A ratio decreases with substantial relaxation impairments. However, it can rise again with the progression of diastolic dysfunction, during a ‘pseudo-normalisation’ phase, when left atrial pressure significantly increases^34^. Furthermore, the E/A ratio is technically challenging to obtain in the BPH mice as they are tachycardic (> 450 bpm) therefore resulting in the fusion of the E and A waves. Since the A-wave cannot be elucidated in some instances (most noticeably in the diabetic BPH mice), the E/A ratio may have been underpowered to detect functional differences between groups. Therefore, it should be analysed in conjunction with secondary parameters such as IVRT to detect functional differences. In addition, partial or full fusion of the two peaks can result in an overestimated E-wave velocity or underestimated E-wave deceleration times^35^. This may explain why there was no apparent increase in deceleration time with diabetes. Nonetheless, markers of impaired cardiac function such as ANP and BNP were upregulated in the diabetic and hypertensive mice.

Endothelial functional studies revealed that co-morbid diabetes and hypertension had minimal impact on ACh-induced endothelium-dependent relaxation, suggesting that these co-morbidities appear to not affect endothelial cells. Co-morbid disease did however significantly enhance PE-induced vasoconstriction, which suggests that vascular impairment observed in this diabetic and hypertensive model is due to vascular smooth muscle cell dysfunction rather than a specific effect on endothelial cells.

Increased oxidative stress is closely associated with maladaptive inflammation in the diabetic and hypertensive setting and can collectively propagate cardiac remodelling and worsening of heart function^36,37^. In the diabetic BPH mice, we observed a significant increase in systemic oxidative stress as well as increased pro-oxidative *p47phox* gene expression in vascular tissue. These markers of oxidative stress were more pronounced in mice that had both co-morbidities. Interestingly, the antioxidant gene NQO-1 was significantly downregulated by hypertension and remained reduced under co-morbid conditions in cardiac tissue, suggesting that cardiac oxidative stress may be increased due to the lack of this antioxidant. Diabetes and hypertension are known to be accompanied by the release of pro-inflammatory cytokines, which induce the expression of adhesion molecules to facilitate leukocyte infiltration into the myocardium. Indeed, we observed an increase in *ccl2* gene expression (encoding MCP-1) in both cardiac and vascular tissue that correlated with an increase in CD45^+^ leukocyte infiltration into the heart. Interestingly, macrophages, as assessed by immunohistochemistry and flow cytometry, did not differ between the groups suggesting that other leukocyte populations may be infiltrating the heart, including neutrophils, Ly6C^+^ monocytes, T cells and B cells. Several studies have demonstrated that monocytes are mobilised from the spleen following cardiac injury and contribute to various aspects of cardiac remodelling through the cardiosplenic myelopoiesis axis^38–41^, whilst conversely, a splenectomy reversed left ventricular remodelling and inflammation^39^. Neutrophils, Ly6C^+^ cell and B and T cell counts were higher in the spleen of hypertensive mice than normotensive mice, with diabetes having no additive effect, suggesting that in this model hypertension alone increases the potential for splenic mobilisation. On the other hand, the potential for mobilisation of leukocytes from the bone marrow is significantly increased in diabetic normotensive mice as compared to other groups, which aligns with the current literature that diabetes is associated with dysregulation of haematopoiesis^42,43^. Our studies suggest that diabetes and hypertension contribute in varying degrees to the inflammation mediated by leukocytes.

One limitation of this study is that inflammation was only characterised at a single time point (10-weeks of diabetes) using flow cytometry and immunohistochemistry. Hence, analysis at earlier time points is warranted to fully characterise leukocyte profiles and their relationship to cardiac dysfunction. Our analysis at the 10-week time point may explain the marginal changes observed in cardiac inflammatory cell populations at this later time-point, since inflammatory changes may have mostly resolved by the time of end-point analysis.

The use of STZ as a diabetogenic agent is widely accepted but has also been controversial. The major obstacle in using STZ is its direct cytotoxic action on major organs, and its inability of sustaining uniformity, reproducibility and induction of diabetes with minimal animal lethality. Thus, several aspects need to be carefully considered, including the method of STZ preparation, stability, suitable dose, route of administration, diet regimen, animal species with respect to age, body weight, gender and the target blood glucose level required to represent hyperglycemia^27^. Another major challenge is that female mice are more resistant to developing STZ-induced diabetes. In our experience, they require higher doses of STZ to achieve equivalent levels of hyperglycemia compared with male counterparts. Higher doses of STZ are known to lead to kidney and cardiac toxicity^27,44^. Since we were specifically interested in studying the effects of co-morbid diabetes and hypertension on cardiac and renal end-points, we wanted to avoid confounding the data due to toxicity. For these reasons in our characterisation studies, we chose a low-dose approach to avoid cytotoxicity and to focus on male mice. Lack of female data is therefore a limitation of this study. As reported previously, no renal abnormalities were observed^26^ and our current study showed no cardiac abnormalities as assessed by Echocardiographic data.

In summary, we have previously reported on the renal pathology in this diabetic and hypertensive mouse model^26^. We demonstrated that these diabetic and hypertensive mice have increased renal oxidative stress, compromised antioxidant capacity and a decline in kidney function as exhibited by an increase in albuminuria^26^. In the current study, we characterised the cardiac and vascular consequences of co-morbid diabetes and hypertension. By showing enhanced inflammatory, oxidative, and cardiac and vascular dysfunction, we have established a framework for future CVD studies using this model. This model offers comparable outcomes with respect to levels of hypertension achieved compared with widely used Ang II-induced rat, without the need for chemical induction of hypertension. This is advantageous since AngII-induced hypertension in rats is highly variable between experiments and between researchers, making it an unreliable model^12^. Hypertension also begins to decline at the start of the second week after AngII-minipump implantation^12^. In our model, hypertension begins around 4-weeks of age, is elevated during the nocturnal phase and persists for the lifetime of the animal, making this a good model for future pharmacological studies of hypertension and co-morbid disease when combined with STZ-induction.

In conclusion, the characterization of the diabetic BPH mouse facilitates the study of two interlinked co-morbidities, diabetes and hypertension, that can be modelled simultaneously. Furthermore, it enables both renal and CVD analyses to be performed in the same model. It is expected that this mouse model will enable the analysis of therapeutic targets unique to co-morbid diabetes and hypertension, thereby facilitating novel therapeutics to emerge in the future.

## Supplementary Information

Supplementary Information.

## Data Availability

The datasets used and/or analysed during the current study are available from the corresponding author on reasonable request.

## Abbreviations

Ach: Acetylcholine
Ang-II: Angiotensin II
BPH: Blood pressure high
BPL: Blood pressure low
BPN: Blood pressure normal
CTGF: Connective tissue growth factor
IL-1β: Interleukin-1β
IVRT: Isovolumic relaxation time
LV: Left ventricular
MCP-1: Monocyte chemoattractant protein 1
NPPA: Atrial natriuretic peptide
NPPB: Brain natriuretic peptide
PE: Phenylephrine
RT-PCR: Real time polymerase chain reaction
SHR: Spontaneously hypertensive rat
STZ: Streptozotocin
TNF-α: Tumour necrosis factor-α
TGF-β: Transforming growth factor β
