# Supplementary material for: Cardiovascular characterisation of a novel mouse model that combines hypertension and diabetes co-morbidities
Source: Sci Rep. 2023 May 30;13:8741. doi: 10.1038/s41598-023-35680-w (PMC10229541; doi:10.1038/s41598-023-35680-w)
Supplement: Supplementary file 1 — Supplementary Information. [file 41598_2023_35680_MOESM1_ESM.pptx]

## Slide 1
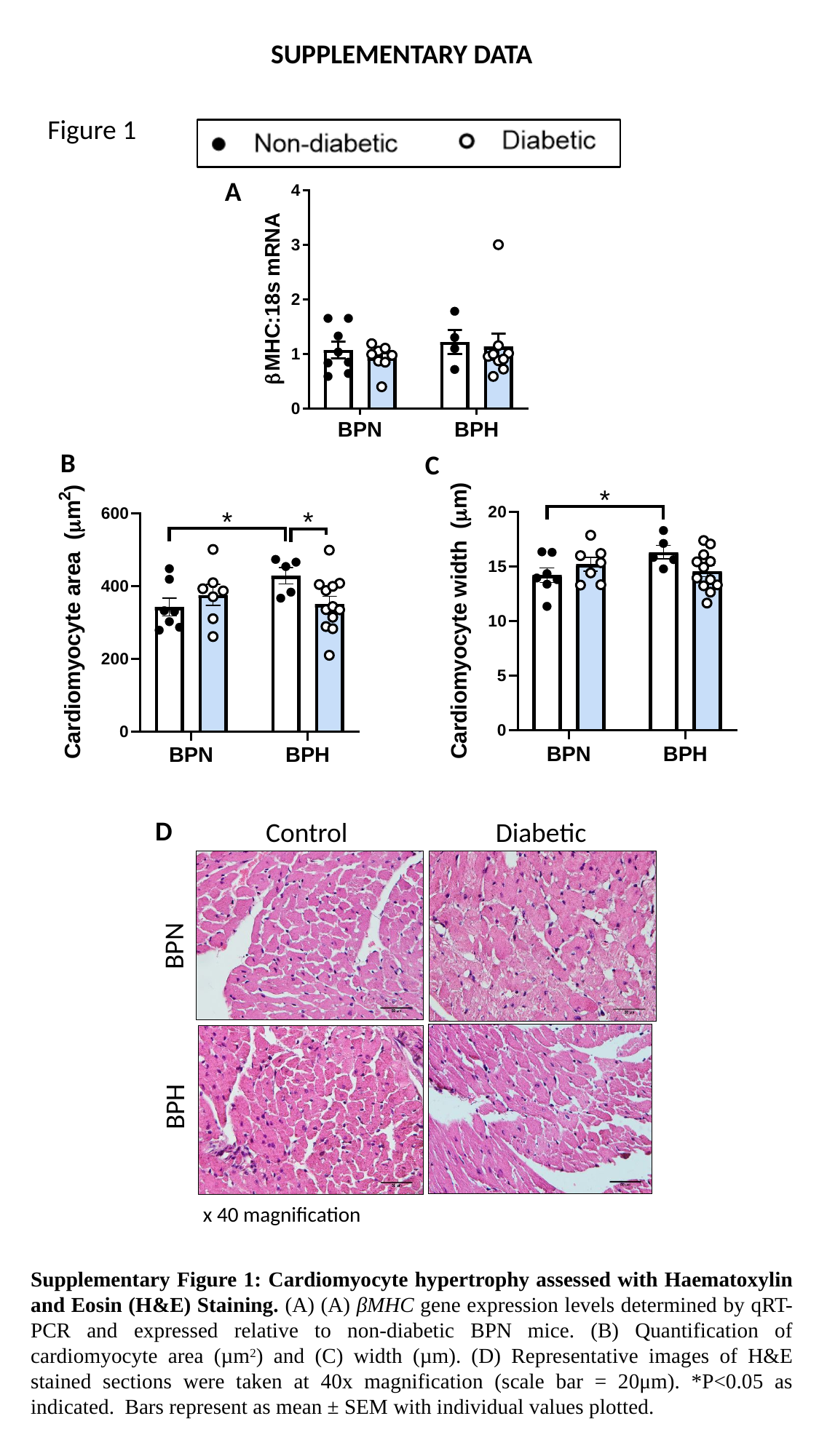

SUPPLEMENTARY DATA
Figure 1
A
B
C
D
Diabetic
Control
BPN
BPH
x 40 magnification
Supplementary Figure 1: Cardiomyocyte hypertrophy assessed with Haematoxylin and Eosin (H&E) Staining. (A) (A) βMHC gene expression levels determined by qRT-PCR and expressed relative to non-diabetic BPN mice. (B) Quantification of cardiomyocyte area (µm2) and (C) width (µm). (D) Representative images of H&E stained sections were taken at 40x magnification (scale bar = 20μm). *P<0.05 as indicated. Bars represent as mean ± SEM with individual values plotted.

## Slide 2
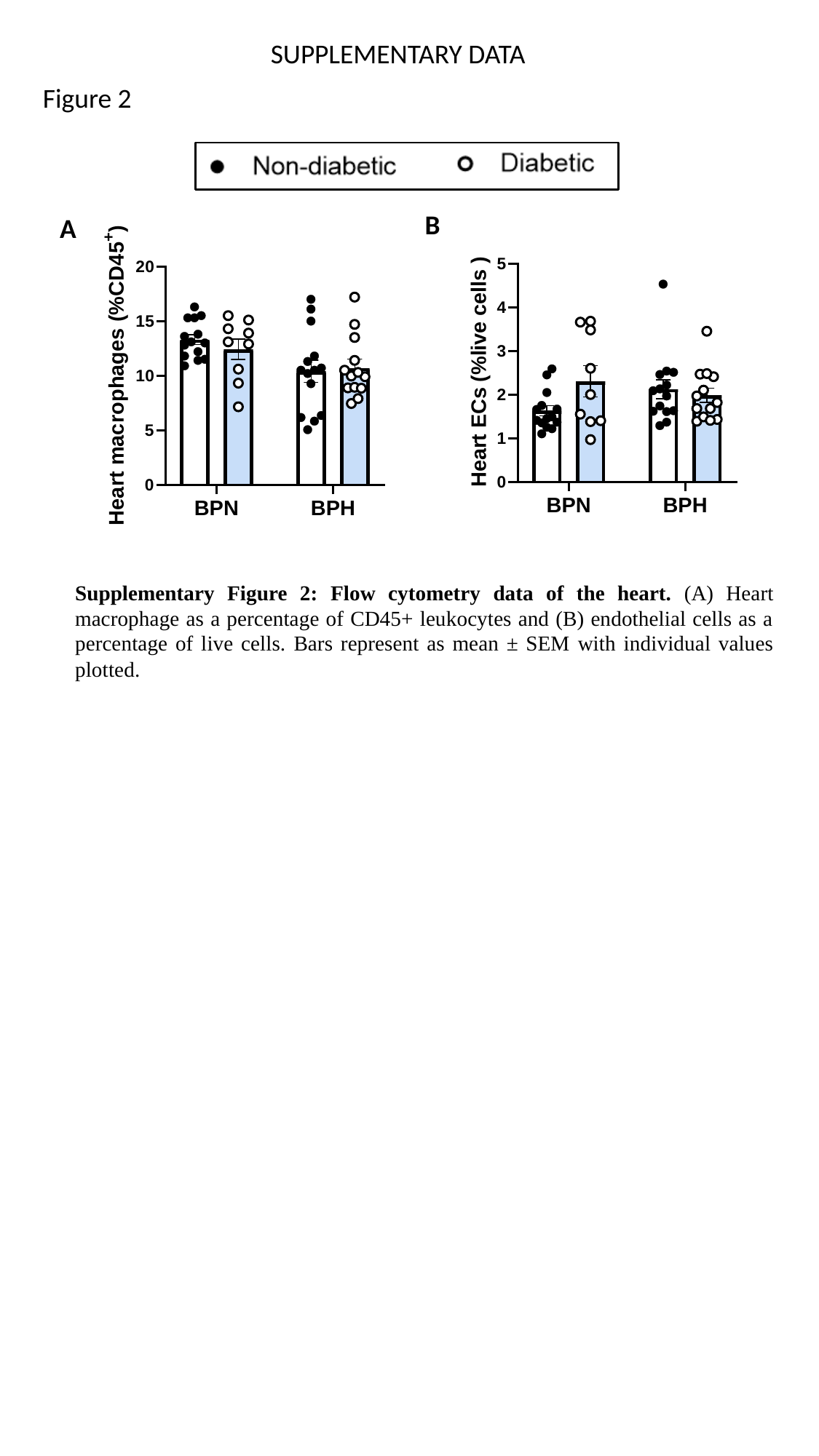

SUPPLEMENTARY DATA
Figure 2
B
A
Supplementary Figure 2: Flow cytometry data of the heart. (A) Heart macrophage as a percentage of CD45+ leukocytes and (B) endothelial cells as a percentage of live cells. Bars represent as mean ± SEM with individual values plotted.

## Slide 3
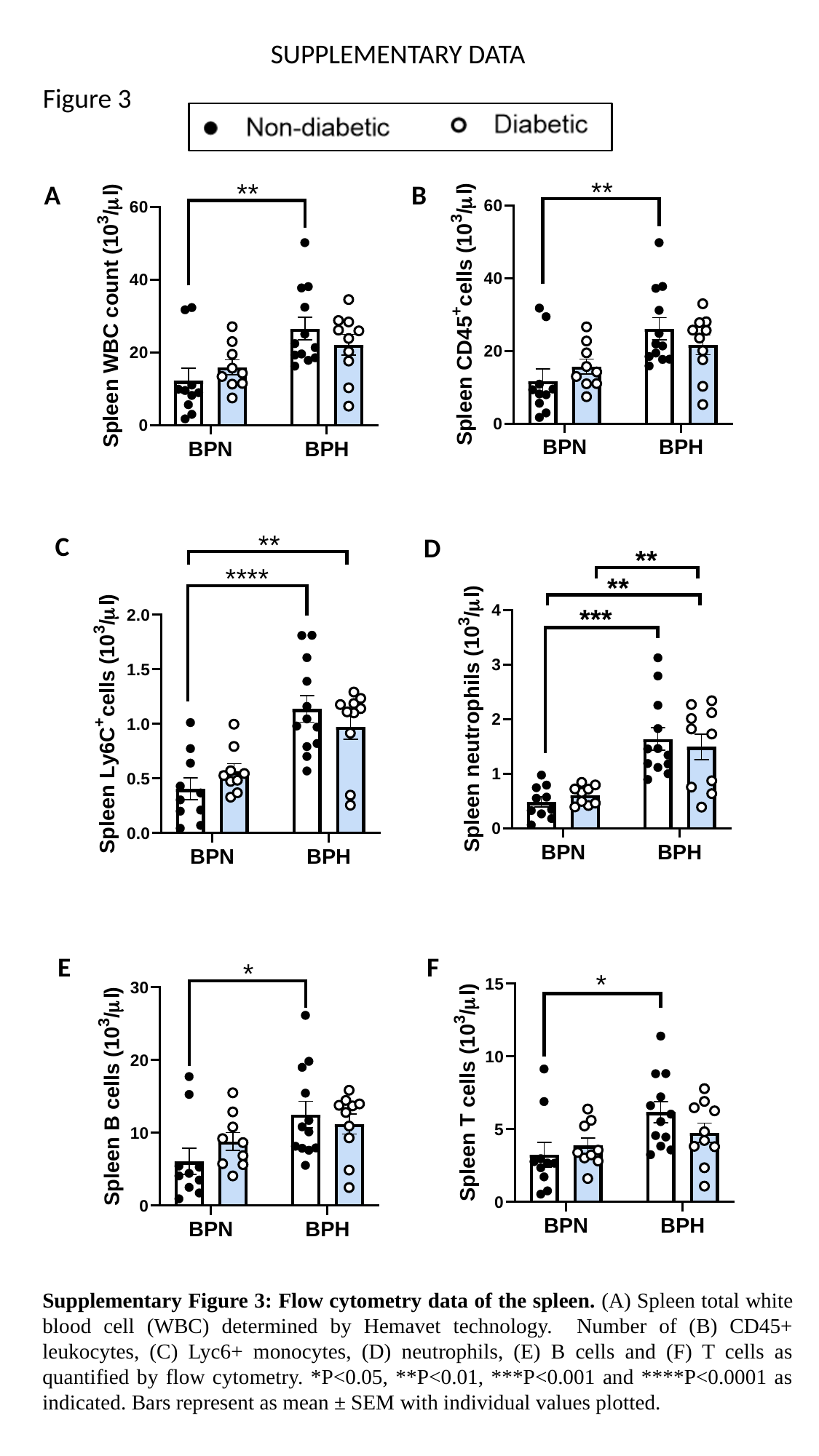

SUPPLEMENTARY DATA
Figure 3
B
A
C
D
F
E
Supplementary Figure 3: Flow cytometry data of the spleen. (A) Spleen total white blood cell (WBC) determined by Hemavet technology. Number of (B) CD45+ leukocytes, (C) Lyc6+ monocytes, (D) neutrophils, (E) B cells and (F) T cells as quantified by flow cytometry. *P<0.05, **P<0.01, ***P<0.001 and ****P<0.0001 as indicated. Bars represent as mean ± SEM with individual values plotted.

## Slide 4
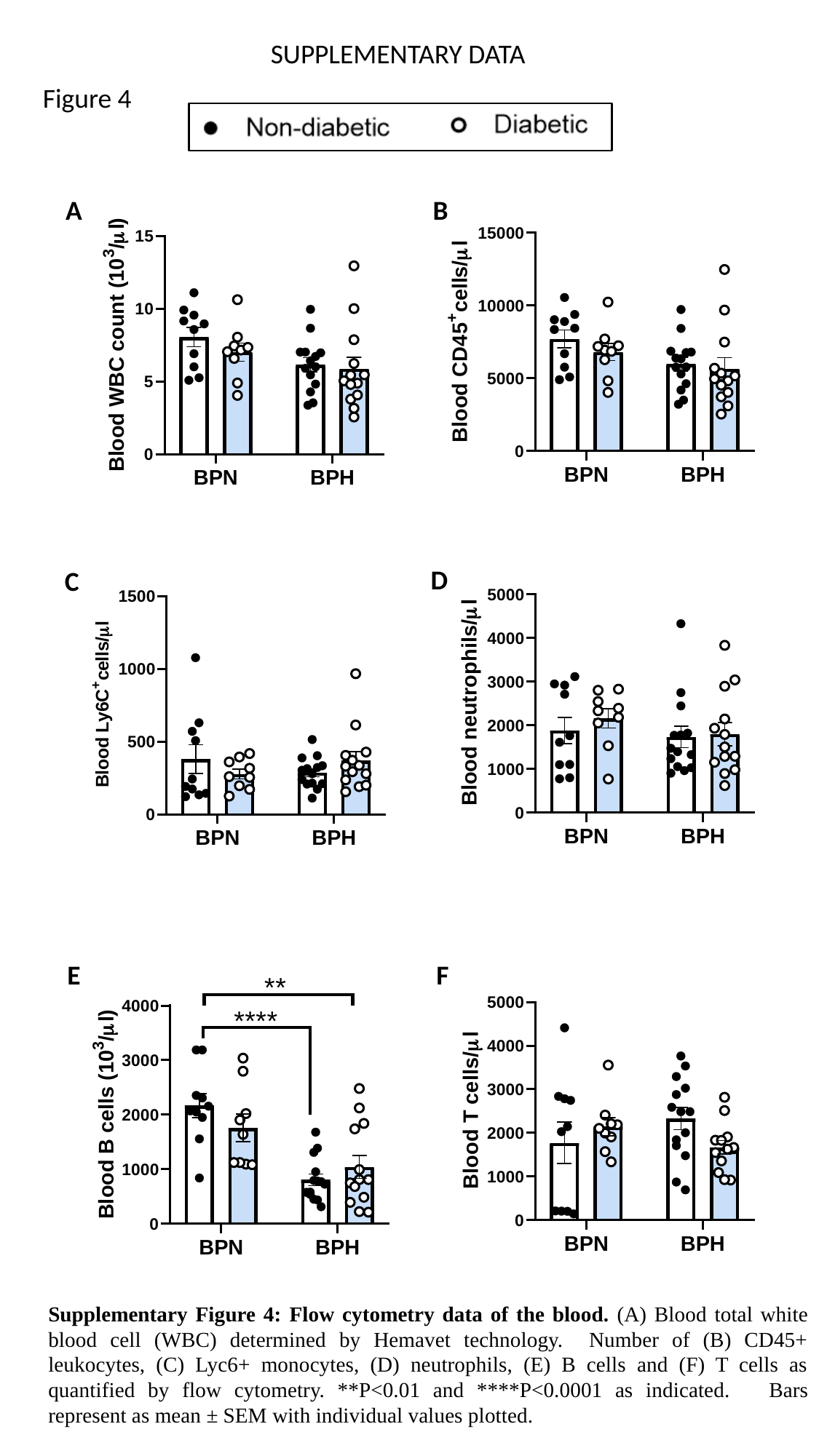

SUPPLEMENTARY DATA
Figure 4
B
A
D
C
E
F
Supplementary Figure 4: Flow cytometry data of the blood. (A) Blood total white blood cell (WBC) determined by Hemavet technology. Number of (B) CD45+ leukocytes, (C) Lyc6+ monocytes, (D) neutrophils, (E) B cells and (F) T cells as quantified by flow cytometry. **P<0.01 and ****P<0.0001 as indicated. Bars represent as mean ± SEM with individual values plotted.

## Slide 5
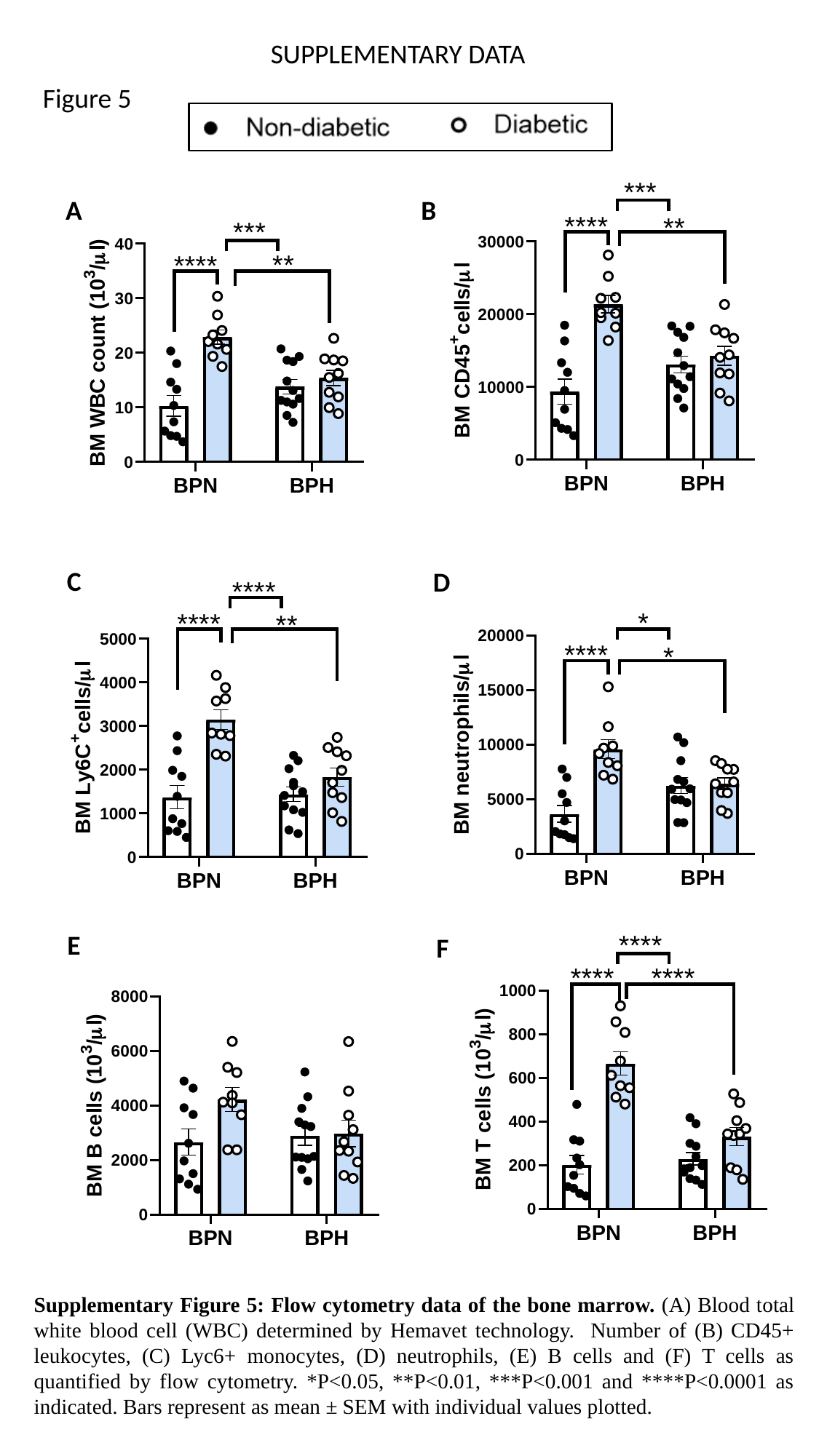

SUPPLEMENTARY DATA
Figure 5
B
A
C
D
E
F
Supplementary Figure 5: Flow cytometry data of the bone marrow. (A) Blood total white blood cell (WBC) determined by Hemavet technology. Number of (B) CD45+ leukocytes, (C) Lyc6+ monocytes, (D) neutrophils, (E) B cells and (F) T cells as quantified by flow cytometry. *P<0.05, **P<0.01, ***P<0.001 and ****P<0.0001 as indicated. Bars represent as mean ± SEM with individual values plotted.

## Slide 6
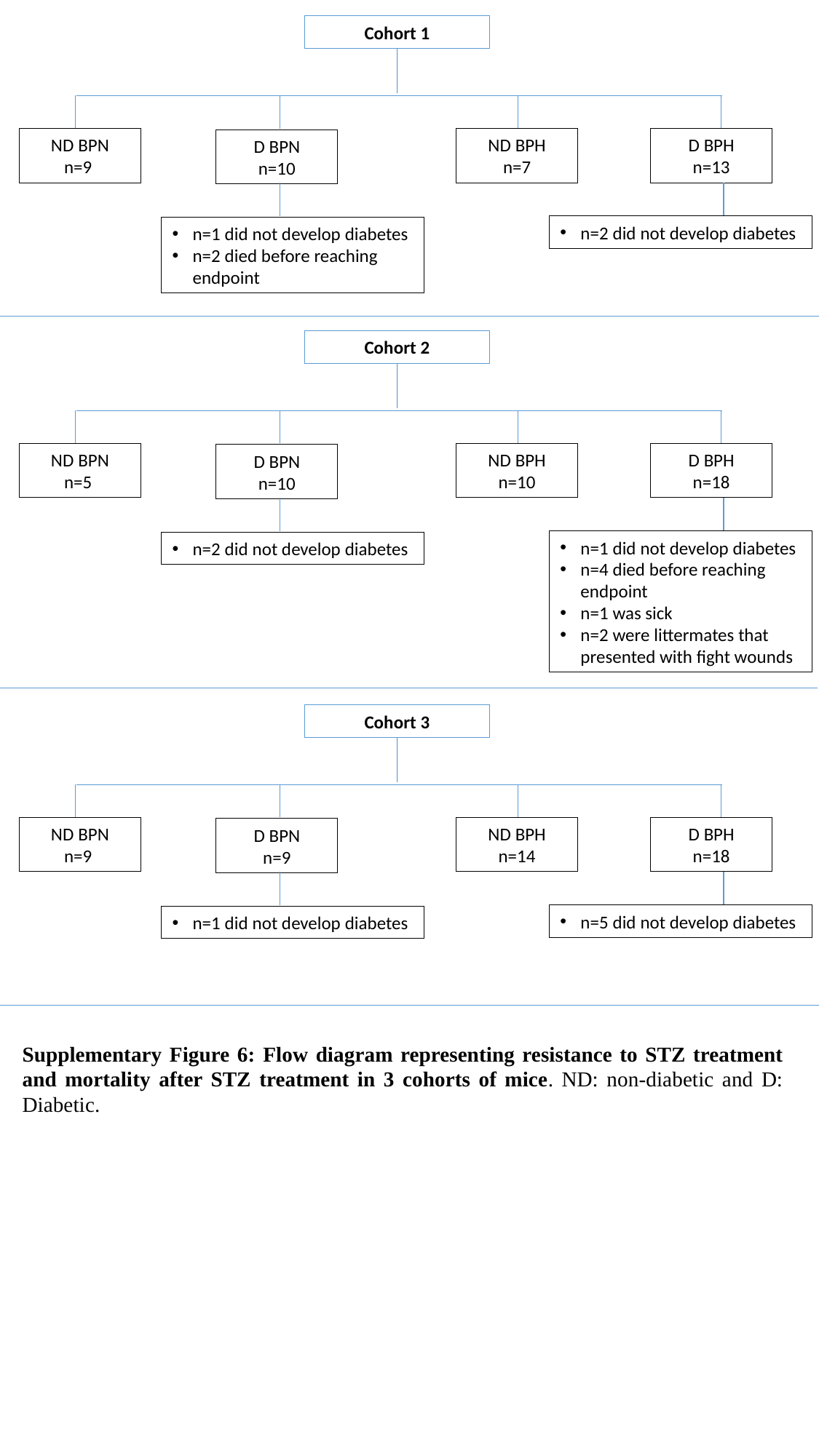

Cohort 1
ND BPN
n=9
ND BPH
n=7
D BPH
n=13
D BPN
n=10
n=2 did not develop diabetes
n=1 did not develop diabetes
n=2 died before reaching endpoint
Cohort 2
ND BPN
n=5
ND BPH
n=10
D BPH
n=18
D BPN
n=10
n=1 did not develop diabetes
n=4 died before reaching endpoint
n=1 was sick
n=2 were littermates that presented with fight wounds
n=2 did not develop diabetes
Cohort 3
ND BPN
n=9
ND BPH
n=14
D BPH
n=18
D BPN
n=9
n=5 did not develop diabetes
n=1 did not develop diabetes
Supplementary Figure 6: Flow diagram representing resistance to STZ treatment and mortality after STZ treatment in 3 cohorts of mice. ND: non-diabetic and D: Diabetic.

## Slide 7
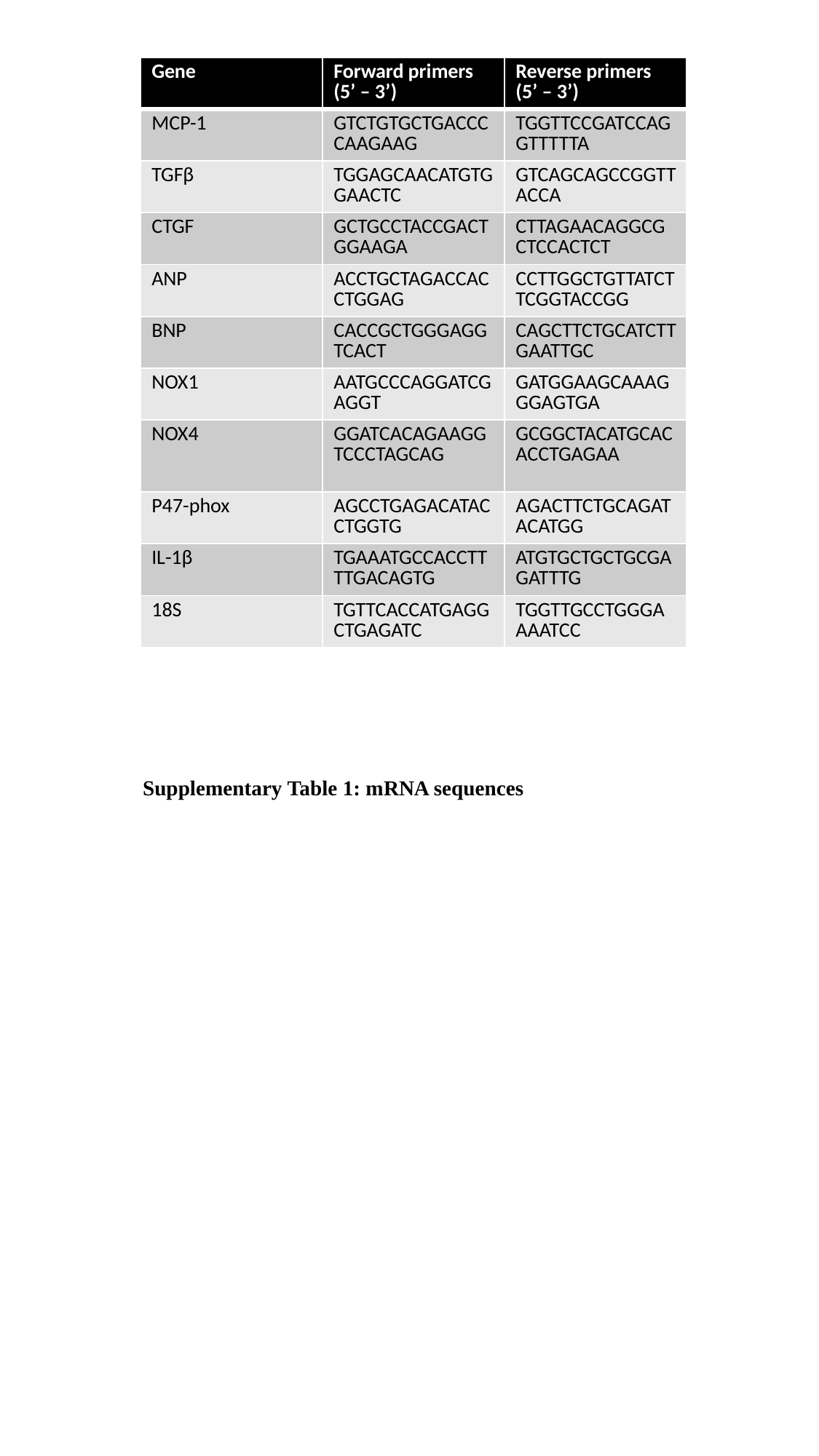

| Gene | Forward primers (5’ – 3’) | Reverse primers (5’ – 3’) |
| --- | --- | --- |
| MCP-1 | GTCTGTGCTGACCCCAAGAAG | TGGTTCCGATCCAGGTTTTTA |
| TGFβ | TGGAGCAACATGTGGAACTC | GTCAGCAGCCGGTTACCA |
| CTGF | GCTGCCTACCGACTGGAAGA | CTTAGAACAGGCGCTCCACTCT |
| ANP | ACCTGCTAGACCACCTGGAG | CCTTGGCTGTTATCTTCGGTACCGG |
| BNP | CACCGCTGGGAGGTCACT | CAGCTTCTGCATCTTGAATTGC |
| NOX1 | AATGCCCAGGATCGAGGT | GATGGAAGCAAAGGGAGTGA |
| NOX4 | GGATCACAGAAGGTCCCTAGCAG | GCGGCTACATGCACACCTGAGAA |
| P47-phox | AGCCTGAGACATACCTGGTG | AGACTTCTGCAGATACATGG |
| IL-1β | TGAAATGCCACCTTTTGACAGTG | ATGTGCTGCTGCGAGATTTG |
| 18S | TGTTCACCATGAGGCTGAGATC | TGGTTGCCTGGGAAAATCC |
Supplementary Table 1: mRNA sequences
